# Genome-Wide Identification of Powdery Mildew Resistance in Common Bean (*Phaseolus vulgaris* L.)

**DOI:** 10.3389/fgene.2021.673069

**Published:** 2021-06-22

**Authors:** Papias H. Binagwa, Sy M. Traore, Marceline Egnin, Gregory C. Bernard, Inocent Ritte, Desmond Mortley, Kelvin Kamfwa, Guohao He, Conrad Bonsi

**Affiliations:** ^1^Integrative Biosciences (IBS), Ph.D. Program, Tuskegee University, Tuskegee, AL, United States; ^2^Department of Agricultural and Environmental Sciences, Tuskegee University, Tuskegee, AL, United States; ^3^Department of Plant Sciences, School of Agricultural Sciences, University of Zambia, Lusaka, Zambia

**Keywords:** genome, crop improvement, genotyping, loci, molecular breeding, GWAS

## Abstract

Genome-wide association studies (GWAS) have been utilized to detect genetic variations related to several agronomic traits and disease resistance in common bean. However, its application in the powdery mildew (PM) disease to identify candidate genes and their location in the common bean genome has not been fully addressed. Single-nucleotide polymorphism (SNP) genotyping with a BeadChip containing 5398 SNPs was used to detect genetic variations related to PM disease resistance in a panel of 211 genotypes grown under two field conditions for two consecutive years. Significant SNPs identified on chromosomes Pv04 and Pv10 were repeatable, ensuring the phenotypic data’s reliability and the causal relationship. A cluster of resistance genes was revealed on the Pv04 of the common bean genome, coiled-coil-nucleotide-binding site–leucine-rich repeat (CC-NBS-LRR, CNL), and Toll/interleukin-1 receptor-nucleotide-binding site–leucine-rich repeat type (TIR-NBS-LRR, TNL)-like resistance genes were identified. Furthermore, two resistance genes, *Phavu_010G1320001g* and *Phavu_010G136800g*, were also identified on Pv10. Further sequence analysis showed that these genes were homologs to the disease-resistance protein (RLM1A-like) and the putative disease-resistance protein (*At4g11170.1*) in *Arabidopsis*. Significant SNPs related to two LRR receptor-like kinases (RLK) were only identified on Pv11 in 2018. Many genes encoding the auxin-responsive protein, TIFY10A protein, growth-regulating factor five-like, ubiquitin-like protein, and cell wall RBR3-like protein related to PM disease resistance were identified nearby significant SNPs. These results suggested that the resistance to PM pathogen involves a network of many genes constitutively co-expressed.

## Introduction

Common bean (*Phaseolus vulgaris* L.) is a notable legume species among the pulse crops that play a major role in addressing global food security, environmental challenges, and health diets (Calles, 2016). However, the productivity of this crop is severely hampered by Powdery mildew (PM) disease, which is one of the most ubiquitous plant diseases, and it infects a variety of legumes, including common bean. PM disease in common bean is caused by the *Erysiphe polygoni* DC pathogen responsible for extensive damage and significant yield losses, up to 69% loss prior to flowering under the environment of warm temperatures (20–24°C) and high humidity, as well as shade environment (Rezende et al., 1999; Schwartz et al., 2005). Because it is an airborne disease, accurate identification and appropriate responses are critical to effectively preventing the spread of PM and minimizing the significant yield losses and the quality of edible seeds.

Initial symptoms appear as small and white talcum-like spots, which are most commonly seen on the upper surface of leaves (Murube et al., 2017). As the symptoms develop, infected leaves gradually curl downward and change color from pale yellow to brown, and ultimately abscise. Under severe conditions, the entire leaves and plants are covered by white cottony mycelia (Pernezney and Stall, 2005), which inhibit the photosynthetic process (Yamashita, 2019) and decrease the rate of photosynthetic carbon dioxide assimilation (Magyarosy et al., 1976).

Several strategies are used to control PM disease, including the application of fungicides, adjusting planting dates to synchronize with periods of maximum sunlight exposure and the adoption of good cultural practices. However, these control methods are expensive and not sustainable. For instance, fungicide treatments may not be effective in minimizing pathogen accumulation (Buruchara et al., 2010). The development of resistant bean varieties is the most economical, efficient, and ecological approach for managing this disease-causing pathogens (Trabanco et al., 2012). Screening sources of resistance and studying its inheritance in common bean genotypes have shown that resistant genotypes carry different resistance genes to the PM disease (Schwartz et al., 1981; Trabanco et al., 2012; Campa and Ferreira, 2017; Murube et al., 2017).

Recently, susceptibility (S) genes have been used as an alternative source for PM disease resistance. The mildew resistance locus (Mlo) is such S genes that promote the pathogen proliferation by suppressing the immune system; therefore, they act as negative regulators of immunity (Pavan et al., 2010; Langner et al., 2018). Loss-of-function studies of some mlo genes have conferred a durable and broad-spectrum, recessively inherited resistance in Arabidopsis (Consonni et al., 2016), cucumber (Nie et al., 2015), tomato (Zheng et al., 2016; Andolfo et al., 2019), and grapevines (Pessina et al., 2016). The comparative genomics approach revealed that five Mlo loci in the common bean genome were clustered in clade V along with Arabidopsis orthologs underlying PM resistance (Rispail and Rubiales, 2016). However, the functionality of these Mlo loci has not been validated yet.

The genome-wide association study (GWAS) is a molecular tool used to identify specific genomic regions or loci governing simple to complex traits. GWAS can be used to determine if a genomic variant is associated with a trait of interest using either germplasm, segregation population, or a collection of diverse genotypes (Challa and Neelapu, 2018; Liu and Yan, 2019). Various candidate genes or quantitative trait loci responsible for traits of interest have been identified using the GWAS approach. For instance, in common bean, several studies have identified candidate genes and their genomic regions associated with different traits, such as bruchid resistance (Tigist et al., 2019); symbiotic nitrogen fixation (Kamfwa et al., 2015a); agronomic traits (Kamfwa et al., 2015b); drought tolerance (Hoyos-Villegas et al., 2017); and anthracnose, angular leaf spot, and Fusarium wilt diseases (Perseguini et al., 2016; Leitão et al., 2020). This study collected several common bean genotypes from different resources to provide enough recombination frequency in the natural population. The BeadChips derived from the common bean were used to cover the genome of the common bean. Therefore, the aim was to reveal the association between genomic regions and PM resistance data using the GWAS approach and identify putative resistance genes.

## Materials and Methods

### Plant Materials and Field Experiment

Seeds of 211 common bean accessions were originally sourced from different germplasm centers (Kamfwa et al., 2015b). Initially, 184 accession seeds were acquired from the common bean germplasm repository at International Center for Tropical Agriculture (CIAT)^1^, and the remaining 27 were derived from Tanzania Agricultural Research Institute (TARI)^2^ Selian center where they were originally collected from Ethiopia (12), Kenya (10), Tanzania (3), and Rwanda (2). These genotypes represented two gene pools based on their origin centers, Mesoamerican origin in Southern Mexico and Guatemala and Andean origin in Peru and Columbia (Kamfwa et al., 2015b). The experimental materials were planted in two locations in northern Tanzania: Selian Agricultural Research Institute (SARI) at a medium altitude of 1407 m ranging between S03°21.690′ and E36°37.879′ and Tanzania Coffee Research Institute in Lyamungo at a low altitude of 992 m between S03°19.905′ and E037°14.067′ in 2017 and 2018. Experiments at each location in each year were laid down in an Incomplete Block Design with two replications for 211 genotypes. The plot size was 3.2 m long and 1.5 m wide with four rows per plot, spaced 0.5 and 0.2 m under rainfed conditions. Data were collected from the middle two rows.

### Phenotypic Data Analysis

The PM disease severity scores were evaluated using a 1–9 scale with 1 being non-pathogenic and 9 pathogenic. Following the scoring rubric of CIAT (Van Schoonhoven and Pastor-Corrales, 1987; Ciat-Kawanda, 2013), the evaluation of the score was conducted twice, i.e., at R-6 and R-8 developmental stages, and the mean of the two evaluations was used for downstream analyses. Analysis of variance (ANOVA) was used to test the interactions of genotype × environment, genotype × season, and genotype × environment × season. Protected least significant differences (LSD) of *p* = 0.05 were used for comparison of genotypes (Clewer and Scarisbrick, 2001; Liu et al., 2008). For each accession, the mean of disease scores obtained from two replications and two environments in each year was used for the phenotype–genotype association study.

### Genotyping Data Analysis

Fifteen seeds per genotype were shipped from Tanzania to Tuskegee University through USDA-Animal and Plant Health Inspection Services (APHIS) with permit number P587-180801-005 and phytosanitary certificate number 00310248 as per import/export plant material regulations. Seeds were planted in a pot with a diameter of 15 cm in the greenhouse at the George Washington Carver Agricultural Experiment Station (GWCAES) of Tuskegee University Research Center, Alabama. Due to germination failure in five accessions, DNA samples were extracted from trifoliate leaves of 206 accessions using Wizard^®^ Genomic DNA Purification Kit from Promega Corporation (Madison, WI, United States). The concentration of each DNA sample was determined using NanoDrop Spectrophotometer (NanoDrop 2000, Thermo Fisher Scientific, Waltham, MA, United States), and the quality of DNA samples was also assessed on 0.8% agarose gel. These 206 DNA samples were genotyped at Heflin Center Genomic Core Lab at the University of Alabama in Birmingham, AL, using the next-generation sequencing (NGS) method of Illumina BARCBean6K_3 BeadChip with 5398 Single-nucleotide polymorphism (SNP) markers (Illumina, San Diego, CA, United States) (Song et al., 2015). All markers were distributed across 11 pairs of common bean chromosomes (2*n* = 22), and the Illumina BeadStation 500G was used to scan the BeadChips. The SNP calling was conducted with the genotyping modules of the 2018 GenomeStudio v2.0.4 open software.

### Population Structure Analysis

The Bayesian model-based clustering method was applied using STRUCTURE 2.3.4 software (Pritchard et al., 2000) to determine the population in this collection. The admixture model with independent allele frequencies without prior population information was used for simulation. The STRUCTURE software was set at a burn-in period length of 50,000, and after burn-in at 50,000, Markov chain Monte Carlo (MMC) repetition was set five times. For joint inference of the population substructure, the kinship (K) was set at a range of 1 to 10 with five iteration runs for each kinship. The ideal number of subpopulations was determined using the K method (Evanno et al., 2005) implemented in the HARVESTER software (Earl and Von Holdt, 2012).

### Marker–trait Association Analysis

Filtering the monomorphic SNP markers and ones with minor allele frequency (MAF) <2% with 6.4% SNPs missing, 5052 SNP markers were retained for population structure analysis and association analyses with TASSEL 5.0 software. The following mixed linear model (MLM) was used:

Y=γ⁢α+ρ⁢β+k⁢μ+ε

where Y is the phenotype of each genotype; γ is the fixed effects of the SNP; ρ is the fixed effect of population structure from principle component analysis results; k is the random effect of kinship relative; and ε is the term error under normal distribution with mean = 0 and variance δ2. The statistical model was used to test for trait–marker associations (Mamidi et al., 2011).

### BLAST Analysis

Significant SNPs were identified using the Manhattan plots for two bean-growing seasons. There are strong positive associations between broad-sense heritability and −log_10_(*p*-value), and significant threshold values are cut off as ∼5 of −log_10_(*p*-value) for the heritability of 70% in soybean (Kaler and Purcell, 2019). To identify the most significant SNPs associated with PM resistance, the cutoff value of −log_10_(*p*-value) was set at five based on 66–71% heritability of PM resistance in common bean (Rezende et al., 1999). To further determine the significant SNPs in multiple tests, the false discovery rate (FDR) was set at α = 0.05 for the calculation of the adjusted *p*-value using the Benjamini–Hochbery test (Benjamini and Hochberg, 1995). SNPs are significant if *p*_i_ < (i/k)α, where *p*_i_ is the *p*-value, *k* is the size of tests, and (i/k)α is an adjusted *p*. The coding genes within the interval of significantly associated SNPs were used as queries to search for putative homologous proteins in the Ensembl Plants release 46 version^3^ (Howe et al., 2020). The coding genes were annotated as candidate genes when they were homologous with genes, kinases, and transcription factors with nucleotide identity >90% and associated with disease resistance. The location of coding genes hitting the putative proteins was used as the position of candidate resistance genes on the corresponding chromosomes.

## Results

### Phenotyping for PM Disease

**FIGURE 1:**
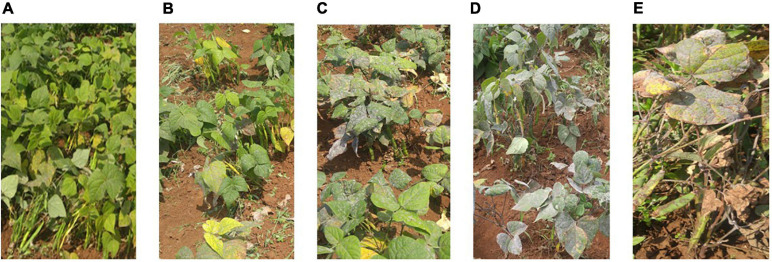
Different disease severity scores evaluated in the field condition. Photos **(A–E)** showed phenotypes with different scales 1, 3, 5, 7, and 9 of the disease severity, respectively.

**FIGURE 2:**
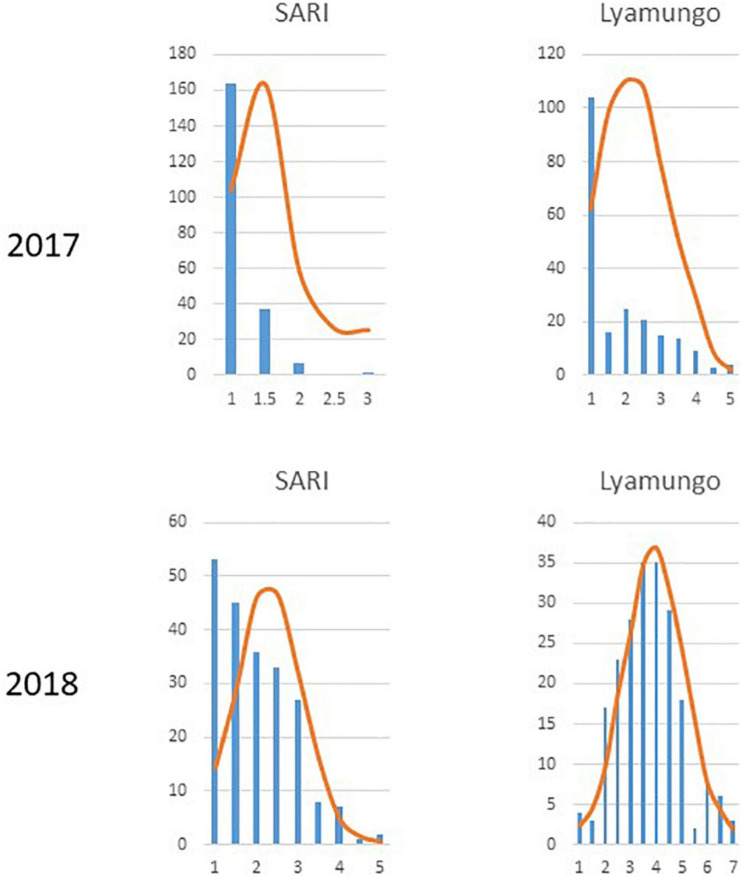
Distribution of phenotypic data across 211 common bean genotypes at each of two locations in 2017 and 2018.

Trial sites were selected for their ideal experimental environments for testing PM disease-resistant traits. They provided higher disease pressure due to consecutive bean growth for more than 5 years resulting in the accumulation of *E. polygoni* inoculum for several seasons. The higher rainfall (>1000 mm) and higher relative humidity (>80%) also created a conducive environment for disease development during these two bean-growing seasons (Binagwa et al., 2020). The environments were used to fully assess the PM resistance potentials of common bean genotypes in nature. Different disease severity scores assessed in the field condition are shown in Figure 1. As a result, highly significant *(p* < 0.001) differences of the resistant trait were observed among the 211 genotypes for PM at two locations in the 2017 and 2018 growing seasons (Binagwa et al., 2020). Also, high significant interactions of genotype × environment and genotype × season were observed, indicating that PM disease resistance is affected by environments (Table 1). Disease severity was higher in 2018, with a maximum disease severity score of nine compared to 2017 with a maximum disease severity score of six among 211 genotypes due to the higher humidity in 2018 compared to 2017. Also, the comparison of the two different locations showed less infection occurrence in SARI (high altitude) than Lyamungo (low altitude), resulting in the distribution of phenotypic data skew toward the resistance (Figure 2). PM’s disease severity under natural infection was negatively correlated with the yield at *r* = −0.24 (data not shown), indicating that PM had a significant effect on the yield of common bean.

**TABLE 1:** Analysis of variance in the collection of common bean genotypes for PM disease.

Source of variation	*df*	SS	MS	*p-Value*
Replication	1	29.4603	29.4603	
Genotype (*G*)	210	668.3436	3.1826	<0.001***
Environment (*E*)	1	624.8395	624.8395	<0.001***
Season (*S*)	1	779.3892	779.3892	<0.001***
GxE	210	337.5355	1.6073	<0.001***
GxS	210	292.9858	1.3952	<0.001***
ExS	1	105.0006	105.0006	<0.001***
GxExS	210	224.8744	1.0708	0.093 ns
Residual	843	785.0397	0.9312	
Total	1,687	3847.4686		

df, degree of freedom; SS, sum of square; MS, mean square; ***, highly significant; ns, not significant. Significant level (*p* = 0.05).

### Population Structure

**FIGURE 3:**
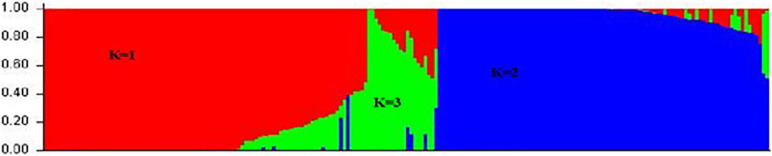
Population structure of 206 common bean genotypes with 5,052 SNP markers, whereby red represents the Mesoamerican gene pool (*K* = 1), blue represents the Andean gene pool (*K* = 2), and green represents an admixture of the two pools (*K* = 3).

The genetic relationship was revealed in the collection of 206 common bean accessions using STRUCTURE software. The genetic population structure was captured by describing the molecular variation in each subpopulation using a separate joint probability distribution over the observed sequence sites or loci based on the Bayesian model. The model was used for association analysis to reduce false associations due to the unequal distribution of alleles among subpopulations. The model grouped 206 genotypes into three clusters (*K* = 3). The first cluster (*K* = 1) consisted of 113 genotypes belonging to the Mesoamerican gene pool; the second cluster (*K* = 2) consisted of 72 genotypes belonging to the Andean gene pool; and the third cluster (*K* = 3) consisted of 21 genotypes belonging to the admixture gene pool (Figure 3). Since this collection included some breeding lines derived from different parents’ sources, there was a possibility that offspring combined the genetic background from both Mesoamerican and Andean gene pools leading to these hybrids in the third cluster. The result suggested that these subpopulations were associated with their genetic background. Interestingly, statistical analysis of disease severity scores based on genotypes in three structure groups showed that the PM resistance was significantly different (*p* < 0.01) between two gene pools, i.e., genotypes of the Mesoamerican gene pool show significantly higher resistances than those from the Andean gene pool. However, genotypes in the admixture group have similar resistance to either gene pool depending on the environment and the season, suggesting that the expression of the resistant trait in admixture genotypes was more easily influenced by the environment.

### Marker–trait Associations for PM Disease

**FIGURE 4:**
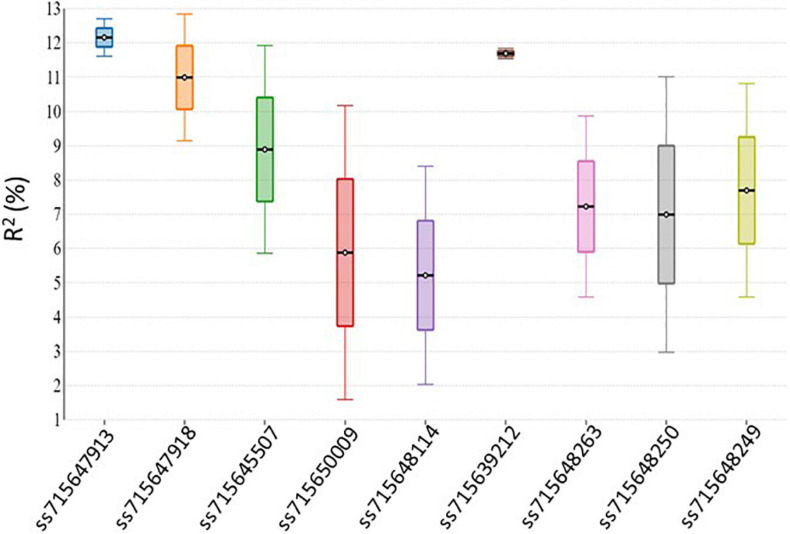
Significant SNPs account for the percentage of phenotypic variation (*R*^2^) in 2 years.

**FIGURE 5:**
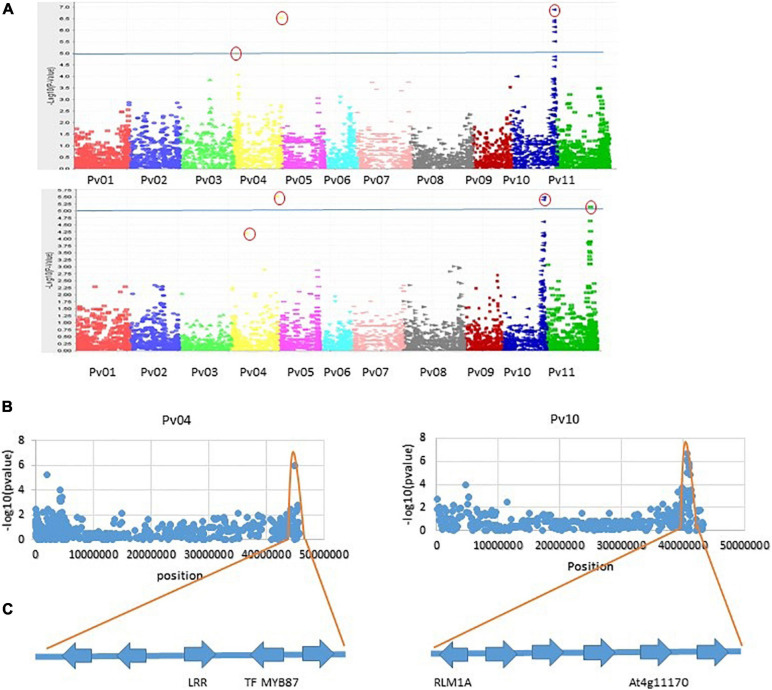
**(A)** Manhattan plots of two seasons for PM disease in 206 collections of bean genotypes using 5052 SNP markers. The horizontal line is the cutoff for significance, and a small red round indicated the most significant markers. **(B)** Distribution of SNPs on Pv04 and Pv10 and the range of SNP peaks. **(C)** Four putative genes identified based on the most significant SNPs and the highest contribution to phenotypic variation.

Genome-wide association analysis of the resistance to PM disease showed comparable results of significant SNPs between the 2-year data. The most significant markers linked to PM resistance were located on Pv04 and Pv10 for both years, indicating the data set’s reliability. A total of nine SNPs were detected as association with common bean PM resistance on Pv04, Pv10, and Pv11 (Table 2). Among them, the SNP (ss715647913) on Pv10 was the most significant (*p* = 4.48 × 10^–07^ and *p* = 2.46 × 10^–05^), contributing the highest phenotypic variation of 12.7 and 11.61% in 2017 and 2018, respectively (Table 2 and Figure 4), while the SNP (ss715639212) on Pv04 represented 11.54 and 11.84% of phenotypic variation in 2017 and 2018, respectively. These two peaks of significant SNPs were located in the range of 45.01–45.02 Mb on Pv04 and 40.57–41.13 Mb on Pv10 (Figure 5). However, the rest of SNPs were significant in 1 year but not significant in the other year. For example, the SNPs located between 2 and 4 Mb on Pv4 were significantly associated with the resistance only in 2017, while the SNPs located between 43.67 and 43.79 Mb on Pv11 were significant only in 2018 (Table 2).

**TABLE 2:** Marker–trait association showing significant SNPs, chromosome position, and contribution to phenotypic variation for powdery mildew disease in 2017 and 2018.

SNP*	Chr.	Position (bp)	2017				2018			
			*p*-Value	Adj. *p*	BH sig.	*R*^2^ (%)	*p*-Value	Adj. *p*	BH sig.	*R*^2^ (%)
ss715647913	10	40,571,646	4.48^–07^	4.71^–05^	Yes	12.7	2.46^–05^	6.6^–05^	Yes	11.61
ss715647918	10	40,624,302	3.76^–07^	3.77^–05^	Yes	12.84	2.66^–04^	2.07^–04^	No	9.14
ss715645507	10	40,937,977	1.21^–06^	6.6^–05^	Yes	11.92	5.61^–03^	6.5^–04^	No	5.86
ss715650009	4	2,013,385	1.04^–05^	8.48^–05^	Yes	10.17	2.54^–01^	1.21^–02^	No	1.59
ss715648114	4	4,303,144	8.62^–05^	1.22^–04^	Yes	8.4	1.72^–01^	8.5^–03^	No	2.03
ss715639212	4	45,014,435	2.76^–07^	1.88^–05^	Yes	11.54	3.10^–06^	9.42^–06^	Yes	11.84
ss715648263	11	43,673,759	1.60^–03^	5.46^–04^	No	4.58	2.29^–05^	5.65^–05^	Yes	9.87
ss715648250	11	43,773,598	1.15^–02^	1.8^–03^	No	2.97	7.23^–06^	4.71^–05^	Yes	11.01
ss715648249	11	43,792,293	7.00^–03^	1.34^–03^	No	4.58	5.35^–05^	7.54^–05^	Yes	10.81

SNP*, single-nucleotide polymorphism code; Chr., chromosome; *R*^2^, phenotypic variation. BH sig., Benjamini–Hochbery significance.

### Identification of Candidate Genes Associated With PM Resistance

There were 181 coding genes observed within the interval of significantly associated SNPs on Pv04, 46 on Pv10, and 24 on Pv11. Using the BLAST analysis of these coding genes against the protein database in GenBank, nine coding genes nearby or within the peak of significant SNPs were considered as candidate resistance genes for the PM disease (Table 3). On the Pv04, three coding genes located at the telomeres were homologous to resistance genes *RPP13*, *TMV-N*, and *LRR receptor-like serine/threonine protein kinase (LRR-RLK) RPK2*, while two coding genes located at the opposite telomere were homologous to one *LRR-RLK* and the transcription factor (TF) *MYB 87*. On Pv10, two coding genes were homologous with *RLM1A-like* and *At4g11170* putative resistance genes, while on Pv11, two other coding genes on Pv11 were similar with LRR-RLK. The sequences of these candidate resistance genes are listed in Additional file 1.

**TABLE 3:** Identified positional candidate genes linked to powdery mildew disease resistance in common bean.

Candidate gene	Position on Pv chromosome (bp)	Similarity	Gene accession	Gene annotation
*Phavu_004G036200g*	Pv04:4014080–4016937	95%	XM_017584784.1	*RPP13-like*
*Phavu_004G028900g*	Pv04:3126300–3130090	99%	XM_017584233.1	*TMV N-like*
*Phavu_004G037500g*	Pv04:4141435–4145345	99%	XM_014658246.2	*LRR-RLK (RPK2)*
*Phavu_004G173300g*	Pv04:45377489–45382880	94%	XM_028069263.1	*LRR-RLK*
*Phavu_004G173500g*	Pv04:45394112–45395393	100%	XM_014638647.2	*TF-MYB87*
*Phavu_010G1320001g*	Pv10:40237562–40238278	95%	XM_022786275.1	*RLM1A-like*
*Phavu_010G136800g*	Pv10:40920056–40929831	95%	XM_014660831.2	*At4g11170.1*
*Phavu_011G167800g*	Pv11:4379752–43809211	98%	XM_028053325.1	*LRR-RLK*
*Phavu_011G169300g*	Pv11:4398238–43999989	97%	XM_028054653.1	*LRR-RLK*

## Discussion

Disease resistance in plants is a complex trait controlled by quantitative trait loci and influenced by environmental factors. The development of resistant varieties requires the identification of resistance genes as a prerequisite in plant breeding. Moreover, understanding the genetic basis of complex traits is needed for molecular breeding. GWAS is such a powerful approach for dissecting these complex traits and has been applied in many plant species, including *Arabidopsis*, *Vigna unguiculata*, and *V. radiate* (Yuan and Kessler, 2019; Lo et al., 2019; Sokolkova et al., 2020). In this study, we used a natural population collected from different common bean resources that possessed many crossover events. The BeadChip with 5398 SNPs provided enough required marker coverage on a diverse set of 206 accessions. The phenotype–genotype association was repeatable between the 2 years using the GWAS approach, suggesting that candidate gene identification pinned by significant associated SNPs was reliable.

Genome-wide association analysis of genes governing PM resistance resulted in the identification of nine candidate genes located on Pv04, Pv10, and Pv11 in the common bean genome that contains a total number of 28,134 coding genes^3^. Pv04 and Pv10 have the lesser coding genes (<2,000) compared to the rest of the chromosomes.

In this study, nine coding genes nearby or within the peak of significant SNPs were considered as candidate resistance genes for the PM disease located on Pv04, Pv10, and Pv11. A coding gene (*Phavu_004G036200g*) was identified at the telomere of Pv04 (between 4.014 and 4.016 Mb). BLAST analysis revealed that this gene is a homolog of the disease-resistance gene RPP13. The RPP13 resistance gene encodes a coiled-coil-nucleotide-binding site–leucine-rich repeat (CC-NBS-LRR, CNL) type of resistance protein and has been known to confer resistance to fungal diseases in plants, including resistance to downy mildew disease in *Arabidopsis* (Bittner-Eddy et al., 2000; Rose, 2002) and resistance to PM in barley (Wu et al., 2018) and in wheat (Wu et al., 2019). This RPP13-like gene located on Pv04 could play an important role in PM resistance in common bean. Recent studies have identified several resistance genes located on the upper region of Pv04, suggesting the existence of a cluster of resistance genes on this arm of Pv04. Murube et al. (2017) have identified three loci related to PM resistance at the top arm of Pv04 in common bean based on the linkage mapping of three biparental segregation populations. Physically, these three loci were located between 0 and 1.09 Mb speculated from the distance (cM) of flanking DNA markers. An additional candidate gene (*Phavu_004G001500*) related to PM resistance was identified by Campa and Ferreira (2017), located between 0.84 and 2.18 Mb on Pv04, suggesting the existence of a cluster of resistance genes in this region of Pv04. Indeed, in this study, BLAST analysis of coding genes around the RPP13-like gene detected several genes related to the PM resistance within the 2-Mb interval between the two most significant SNPs, including *Phavu_004G020000g* (senescence-associated carboxylesterase 101), *Phavu_004G028900g* (TMV resistance protein N-like), and *Phavu_004G037500g* (LRR-RLK, RPK2). Both TNL and CNL proteins are involved in pathogen recognition but differ in signaling pathways. The TMV resistance protein N is encoded by the Toll/interleukin-1 receptor-nucleotide-binding site–leucine-rich repeat type (TIR-NBS-LRR, TNL) gene (Mestre and Baulcombe, 2006); activation of resistance response involving TNL requires several known general cofactors of disease resistance, including protein kinases (Jin et al., 2003). At the opposite telomere on Pv04, additional genes *Phavu_004G173300g* (LRR-RLK) and *Phavu_004G173500g* (MYB87) were also identified. These genes could be involved in disease response since MYB87 functions as a regulator of genes affecting cell wall organization and remodeling (Fujiwara et al., 2014). It is probable that upon pathogen infection, the constitutive expression of this set of genes located on Pv04 could show the characterization of quantitative resistance trait to the PM disease in common bean. Further investigation is needed to elucidate the molecular mechanism of the cooperation between TNL and CNL pathways. Whether TMV-like genes mediate the resistance to PM disease requires the protein kinase LRR-RLKs to induce the effector-triggered immunity (ETI) or requires alternative spliced transcripts to promote resistance proteins that can specifically recognize the pathogen molecular elicitors (Song et al., 2014).

Based on the 2 years’ data, two other candidate resistance genes, *Phavu_010G1320001g* and *Phavu_010G136800g*, were identified at the bottom arm of Pv10 with 0.6 Mb apart nearby the significant SNPs. The former candidate gene was homologous with the disease-resistance protein RLM1A-like, while the latter hit the putative disease-resistance protein (*At4g11170.1*). The *RLM1A* gene confers resistance in *Arabidopsis* against *Leptosphaeria maculans*, a fungus pathogen, and is involved in the first layer of defense through the callose deposition acting as a temporary cell wall in response to pathogen attack (Staal et al., 2006; Persson et al., 2007; Delgado-Cerezo et al., 2012). The gene *At4g11170* refers to disease-resistance gene *RMG1* (Resistance Methylated Gene 1) (Yu et al., 2013). The expression of this gene is controlled by DNA methylation on its promoter region. The RMG1 promoter region is constitutively demethylated by active DNA demethylation mediated by the DNA glycosylase ROS1 (Yu et al., 2013). Both identified candidate genes encoded the TNL resistance protein and are closely located on Pv10. However, they did not show significant similarity in either DNA or amino acid sequences, suggesting that they were not derived from the duplication event. These two clustered resistance genes may meet the digenic requirement for functional resistance as observed in the RPP2 that consists of a complex of TIR-NB-LRR genes for defense response (Sinapidou et al., 2004).

Additional candidate resistance genes *Phavu_011G167800g* and *Phavu_011G169300g* were detected on Pv11 based on the significant SNPs from the association analysis in 2018. Both genes were annotated as LRR receptor-like serine/threonine protein kinase (*At1g5430* and *At1g56130*, respectively). LRR receptor-like kinases (RLK) represent one of the largest protein families in plant (Gou et al., 2010). The LRR-RLK proteins in plants are involved in the plant signaling pathway regulating pathogenic defense responses (Afzal et al., 2008). The LRR domain of RLK has undergone an accelerated evolution that generated numerous cell surfaces and cytoplasmic receptors to interact with a diverse group of proteins for the specificity of pathogen recognition (Afzal et al., 2008). LRR receptor-like serine/threonine protein kinase was identified as a candidate gene conferring the resistance to apple scab, as demonstrated by Padmarasu et al. (2018). In this study, LRR-RLKs were identified on Pv11 as associated with PM resistance based on 1-year data. Although resistance loci were detected on Pv11 using different genotype materials of common bean (Pérez-Vega et al., 2013; Murube et al., 2017), further study of the interaction between LRR-RLK and PM pathogen will help with understanding the mode of action of these resistance genes. Nevertheless, many genes encoding the auxin-responsive protein, TIFY10A protein, growth-regulating factor five-like, ubiquitin-like protein, and cell wall protein RBR3-like protein were linked to significant SNPs, suggesting that many genes could be involved in the resistance to the PM disease acting in cascade through several layers of defense barriers or in synergy to induce reactions.

As discussed above, nine putative resistance genes were identified within the peak ranges of significant SNPs. However, the reliable significant SNPs are considered when the Benjamini–Hochbery test was significant in both 2017 and 2018. In this study, two significant SNPs, ss715647913, and ss715639212, met the criterion. Two genes LRR-RLK and TF-MYB87 on Pv04 were identified in the peak of ss715647913, while RLM1A and At4g11170.1 on Pv10 were detected in the peak of ss715639212 (Figure 5). These four putative genes also accounted for the highest percentage of phenotypic variation (*R*^2^ > 10%) in both years (Table 2 and Figure 4). Other significant SNPs with their related genes passed the Benjamini–Hochbery test only in either year. For example, RPP13-like, TMV N-like, and RPK2 on Pv04 were identified around SNPs ss715648114 and ss715650009 which were significant only in 2017, while two LRR-RLKs on Pv11 were identified between SNPs ss715648249 and ss715648263, which passed the test only in 2018. Therefore, these genes must be confirmed in different genetic backgrounds or by multiple locations/years. Even though these four reliable candidate genes may play key roles in the resistance, the function of these genes needs to be validated by performing loss-of-function studies for accurate marker-assisted selection.

## Conclusion

The PM disease causes significant yield losses in common bean. Identification of resistance genes can not only provide a resource for the resistance breeding but also facilitate understanding of the mechanism of interaction between host and pathogen. In this study, we used the GWAS approach to identify SNPs associated with PM disease resistance. The most significant SNPs were identified on Pv04 and Pv10. Association of the significant SNPs with the PM disease resistance revealed four putative resistance genes. Results were repeatable in different years, suggesting their reliability. However, other genes were significantly associated with the PM disease resistance only in 1 year, suggesting that a further confirmation under a different genetic background is needed. Nevertheless, our results demonstrated the presence of putative resistance genes and their locations on the common bean genome, which could be utilized for marker-assisted selection. Functional genomic study approaches are needed to validate the role of these putative genes to benefit breeding programs for common bean improvement.

## Data Availability Statement

The datasets presented in this study can be found in online repositories. The names of the repository/repositories and accession number(s) can be found in the article/Supplementary Material.

## Author Contributions

PB, GH, and CB designed the research study and developed the manuscript. ST and KK analyzed the data. ME, GB, IR, and DM commented and corrected the manuscript. All authors proofread and approved the final version.

## Conflict of Interest

The authors declare that the research was conducted in the absence of any commercial or financial relationships that could be construed as a potential conflict of interest.

## Footnotes

1 www.ciat.cgiar.org

2 www.tari.go.tz

3 https://plants.ensembl.org/Phaseolus_vulgaris/Location/Genome?r=11:1-1000
