# Supplementary material for: Genome-Wide Identification of Powdery Mildew Resistance in Common Bean (Phaseolus vulgaris L.)
Source: Front Genet. 2021 Jun 22;12:673069. doi: 10.3389/fgene.2021.673069 (PMC8258261; doi:10.3389/fgene.2021.673069)
Supplement: Supplementary file 1 [file Table_1.DOCX]

Table S1. List of sequences of candidate resistance genes to PM in common bean

>XM_007151255.1 (PHAVU_004G036200g) RPP13-like

ACAAAAGATGTTCCACCATCTTAAAGTCAAGTCATCCTCTGTAGAACATCGTATACTCATCTCATCAATA

ATTGTTACGAACTGAAACTGATTTCTATTAACCCTATCCCTCACCAATTTCTACCATGGCAGCAGAACTT

GTTGGTGGTGCTCTTCTTTCGGCTTTCCTTCAGTTTGCATTCGATAGGCTGGCCTCTCCTCAGGTTATAG

ACTTCTTTCGGAGAAGAAAACTTGATGAGAAACTGCTCGGCAATTTGAACATCATGCTACACTCCATCAA

TGCTCTCGCTGATGATGCTGAACTAAAGCAGTTCACAGATCCACATGTCAAAGCATGGCTTTTTGCGGTC

AAAGAGGCTGTCTTTGATGCAGAGGATCTTTTGGACGAAATAGATTATGAACTCACCAGATGTCAAGTGG

AAGCTGAACTTGAACCTCAAACCATCGCTTACAAGGTATCAAACTTCATCAACTCTACTTTCAGTTCATT

TAACAAGAGAATTGACTTAGGGTTGAAAGAAGTCTTAGAAAAATTAGAATATCTTGCAAGGCAAAAGGGT

GCTCTTGGTTTGAAAGAGTGTACTCATTCTGGTGCTGGATCAGGTAGTAAAGTGCCATCATCTTCTTTGG

TGGTTGAAAGTGTTATTTATGGCAGAGATGCTGACAAAGATTTAATCATTAATTGGCTCACATCGGAAAC

CAATAATCAAAACCAGCCATCAATTCTTTCTATTGTGGGTATGGGTGGGTTGGGTAAAACCACACTCGCT

CAACATGTATACAATGACCCAAAGATTAACAATGCTAAATTTGATACCAAAGCTTGGGTCTGTGTTTCAG

ATCATTTTCATGTTTTGACCGTGACAAAAACTATTCTTGAGGCAATCACTGATAGAAAAGATGACAGTGG

AAACCTAGAAATGGTTCACAAAAAACTGAAACAAAATTTGTTAGGAAAGAAATTTCTTCTTGTATTGGAT

GATGTTTGGAACCAAAGACGAGACGAATGGGAAGCTGTGCAAACTCCTCTTACCTACGGCGCTACTGGAA

GTAGAATTCTTGTGACAACACGTGATGAGAAAGTTGCTTCTAACATGCAATCTAAAGTGCATCGCCTAAA

ACAATTACGAGAGGATGAATGCTGGAACGTTTTTGCAAAACACGCATCAATAGATGATAATCTTGAATTG

AACAATGAGCTAAAGGAGATTGGTAGAAGGATAGTTGAGAAGTGCAAAGGATTACCTTTAGCTTTGAAAA

CAATTGGATGTCTTTTACGCACAAAGTCATCCATTTCAGATTGGAAAAGCATATTGGAAAGCGACATATG

GGACTTACCAAAGGAAGAGAGTGAAATTATTCCTGCGCTATTTCTGAGCTATCACTACCTTCCTTCTCAT

CTTAAACGGTGCTTTGCTTATTGTGCCTTATTCCCTAAAGATTATGAGTTTGTGAAGGAGAAGTTAATTT

TGTTGTGGATGGCCCAAAACTTTCTAGGATGCCCTCAACAGGTTAGACATCCTGAAGAAGTTGGTGAACA

ATATTTCAATGATCTTTTGTCAAGGTCTTTCTTTCAACAATCAATCACCAAAAGGGGTTTTGTCATGCAT

GACCTTCTGAATGATCTAGCAAAATATATTTGTGGGGACATCTGTTTCAGGTTGAGATTTGATAAAGGAA

AATGTATACCAAAAACAACCCGTCATTTTTCATTTGCATTAGATGATGTCAAATGTTTTGATGGTTTCGG

GAGTTTAACTGATGCTAAAAGACTGCGTTCATTTATTTCAATTACAGAAATTGGGAGAACATGCATTGGC

TATTATCCTTGGGAGTTCAAGATTTCAATACATGATGTGTTCTCCAAGTTTAATTTTTTACGTGTTATAT

CTCTCTATTCTTGTTTGGACCTTGTAGAGGTCCCAAATTCTATAGGTAATCTTAAACATCTCCAATCGTT

AGACCTTTCGAAAACTGGGATACAAAAGCTACCCGACTCAACATGTTTGCTCTATAACTTGCTAATACTA

AAGTTGAACTTTTGTGGATATCTGAAGGAGCTGCCATCAAATTTGCATAAACTCACCAAATTACGTTGCC

TGGAGTTTAAAAATACAAAAGTGACAAAGTTGCCAATGGATTTGGGAGAATTAAAGAATCTTCGAGTACT

GAGTACCTTTTTTGTCCATAAAAATAGTGAATTCAGTAATAAACAGCTAGGAGGACTCAATCTTTATGGA

GACCTATCAATTAAGGAGCTGCAAAATATTATGAATTCATCGGGAGCATTAGCAATGGACTTGAAAAACA

AAACACACCTTATGAGGTTAAAGTTAAAATGGAATAAAAATCACACCCCTGATGATCCAAGGAAAGAAAA

GGAACTACTTGAGATTCTGCAGCCCTCCAAACACTTGGAGCGTTTGTCAATCAGAAACTACAAAGGTATA

GAATTCCCAAGTTGGATATTTGATAATTCGTTATCAAATTTGGTGTTCTTACGGTTGGAGGACTGTAAAT

ATTGTCTATGTTTGCCTCCCCTTGGGCTTTTGTCATCTCTGAAGATCCTCAAAATTAGACGGCTTGATGG

AATAGTGAGCATTGGTGCTGAATTTTATGGGACCAACTCTTCATTTACGTCCTTGGAAAGGTTGGAATTC

TACAACATGAAGGAATGGGAAGAATGGGAATGTAAAACTACTTCTTTTCCACGTCTTCAACATCTTTCTG

TGGATCAATGTCCCAAGCTGAAAGGTTTGTCAGAGCAACTTATTCATTTAAAGAAATTATTTATTTCTCA

CTGTGATAAGCTCATCATTAGCGGCATGGACACATCATCGCTTGAACTCTTGGAAATT

>XM_028069263.1 (PHAVU_004G173300g) LRR receptor-like serine/threonine-protein kinase At4g29180 TTCAAATCACATATGTGCACTAATTTGTCTTGAGTAATTAAAAAATGTTGCAGGGACATAAATAATTATC

CATTTTTTTTGTGTAATCACGTTTTGCAAACTGTTATGGGGGCGTGATTCTCTTCCATTTTTGATGAATT

TTTTTTAGAGTTTTAACATTTCAGTGTCTGGTTTTGTCAAAACCAACAAAATGCGTGTCCAAGTTGACAT

GTTAACGTTTGACCCATTATTTTGTTTTTGTTTTTGTTTTTGTTTTTCCACTCTCTAATGTTTCCATGGA

AGTACATGTCTACAAGGATCCGAACAGCCACGGATTTGAAACACGTAATATAAGGTTTGATAGTGTCTTC

TCTGGTTTCCAGCTATGGGAAGTTCGATTGGGTTCATACTGTTGCTGACACTCTGTGTTGTCCTCATAGC

TTTGGTTCAGGCACAACAACAAATAGGCTTTGTAAGCATTGATTGCGGTAGTTCGGAAAATTTGTACACA

GATGAGTCAATTAAAATAATGTACACAGGTGATGGATCATATATACAGAGTGGGGTTATTAAGAACATCT

CCACTGAGTATAATTACCCAAACAATCCCAATCTGCCACAAGTACTCTCAGATCTCAGAAGTTTTCCCAA

CGGAGAAAGGAACTGTTATAGTATAGCAGGTTTGAGAAGTGACAGTTTATATCTGATCAGAGCTTACTTC

TTGTATGGAAACTATGATGGAGAAAACAAGCCTCCAGAGTTTGATCTCTATGTTGATGTCAATTTCTGGT

CAACAGTGAAATTCAGAAATGCCTCGGAGGAACTTGTGCTTGAAATAATCAGTTTGGCACAATCAGATGT

GACATATGTTTGCCTTGTGAACAAGGGAACAGGAACTCCTTTTATCTCAGGATTAGAGCTTAGACCAGTT

AATAGTTCTGTTTACAACACTGAGTTTGGGGAACCTGCTTCGCTAACACTTTTCAAACGATGGGACATTG

GGTCACTCAATGGAAGTGGTAGATACGAGGATGATATTTATGATAGAATCTGGTCCCCTTTCAATTCTTC

ATCCTGGGATTCTGTCAGCACTTCTGAGCCAATAAATGTCAATGGTGATGGCTTTAGACCACCATTTGAA

GTTATTAGGACTGCTGCTAGACCAAAGAATGGCACTGATACTTTGGAGCTTTCTTGGACCCCAGATGATC

CAAGTTGGAAATTTTATGTGTACTTGTACTTTGCTGAAGTGGAGCAGCTTGAGAAAAACCAGCTCAGGAA

ATTCAATATATCTTGGAATGGATCTCCATTGTTTGATTCCATAGTACCTCGCCATTTGTTTGCGACCACC

CTTTCTAATTCAAAATCTTTGGTGGCAAATGAACATCGTATTTCCATACGCAAAACAGAAGATTCAACCC

TTCCACCTATTCTTAATGCAGTTGAGATTTATGTAGTGAAGCTAGATGCACTTGCAACATTTCAACAAGA

TGTTGATGCTATGGTGGATATAAAGGAAAGCTATAAAATTCAAAGAAATTGGATGGGTGATCCATGTGAG

CCAAAGAATCACTCTTGGGAAGGCTTAACATGCAACTATAGCACTTCAGTTCCTCCCAGAATTATATCTC

TGAATATGAGCTCCAGCAGTTTGAGTGGAACAATAACTTCTGCCATTTCCAATCTCTCGTCGCTGGAATC

TTTGGATTTACACAACAATAGCTTAACGGGAGCAGTGCCTGAGTTCTTGGAAGAATTGAAATCCCTTAAA

TATTTGGATTTAAAGGACAATCTATTTTCTGGTTCAGTTCCTGTCACTCTTTTAGAAAGATCAAGGGCTG

GGAAACTTACATTGAGGGTGGATGATAAAAATCTTGGTGGCTCAGGAGGGAGTAGTAATAAAACCGTTAA

AATTGTGGTTCCCATAGTGGTATCAGTATCAGTTATAGTTATATTGGTTGCTTTCATTATCTTTTGGAAA

CATAGAAGAAATGAACGATCAGATGAGGAGATCAGTATGCTGAACAAAAGAGGGAAAAATGTAACAACGA

AGAACTGGCAATACACGTATTCGGAAGTATTGGAAATCACCAACAACTTTGAAAAGGTCATTGGCAAGGG

AGGATTTGGAATTGTGTATAGTGGGCAGATGAGAGATGGCAAAGAAGTTGCGGTTAAGATGCTTTCTCCA

TCATCATCTCAGGGCCCAAAAGAATTTCAAACTGAGGCTGAGCTTTTGATGACAGTTCATCACAAAAATT

TGGTATCCTTCGTTGGTTATTGTGATGATGATAACAAGATGGCTCTCATATACGAGCACATGGTCAATGG

CAGCCTGAAAGATTTTCTCTTTCTCTCTGATGGAAATTCACATTGCTTGAGTTGGGAAAGAAGAATACAG

ATAGCAATCGATGCCGCTGAGGGGTTGGATTACCTACACCATGGTTGCATGCCACCAATAGTACACAGGG

ATGTAAAGTCAGCAAACATTCTTTTAAGTCAAGATTTAGAAGCCAAGATAGCAGATTTTGGCCTCTCCAA

GGAGTTTAGGAAAGATAACCAAAAACAACAATCTCATGTGATTCATACTGATGCTACAAATGACCAATCT

GCAATAATGGGCACAACAGGGTACCTTGACCCAGAGTACTACAAATTAGGGGGGTTGACAGAGAAAAGTG

ACATCTACAGCTTTGGAATTGTTCTACTTGAATTAATCACAGGTCGCCCTGCAATATTTAAAGGCAACAC

AATAATGCACATACTTGAGTGGCTAAGACCTGAGCTTGAAAGAGGAGAATTGAATAAAATTTTAGATCCA

AGGCTCCAAGGAAAATTTGGTGCAAATTCTGGGTGGAGAGCTTTAGGAATAGCATTGCAATGTTCTGCAT

CAACCTCCATTCAGAGACCTACAATGAGTGTTGTGATAGCAGAGTTAAAACAGTGTTTGACAATGGAATC

TCCTAGTAACACTGAAACATTTGTGCCCCCTCCAAAACAAATCTACACTGAATTCTACAGTTCGTCAGAA

GCACATTCTTATGATAGTGAATCTATCACCTATTCTTTCCCAAGATAGCAGTATACATGGGACCATTGGG

ATTCATTAATTAAACTGTAATTTTTTTGTGAAACTGTAAGATGAAAGGCACACTGTTACTGCAACAAATG

GCACTCTTCTGCAGTATTTTTTTGAACAAAATATTTATATTGATTTGGTCTTGTTCAGTAATACTTGGAT

AACTTCAA

>XM_014638647.2 (PHAVU_004G173500g) transcription factor MYB87-like

ACACTGTGTTCTTATAACCACATCTTTTGTGCTCTCTTTTGACATCTTCTCTAAGCCACTATCTTCACCA

AATACATTCACTTTCTCTCTCTGCCATTGATCAATCCTGGTGGTATATTCAGTTCATCAGTGGCCAAGTA

GGCCATGGGAAGGGCTCCATGCTGTGACAAATCTATCGTGAAAAAAGGACCATGGTCTCCTGATGAAGAT

GTCACCCTCAGAAACTACCTTGAGAAACATGGCACTGCTGGAAATTGGATAGCCCTCCCCAAGAAAGCAG

GGCTTAAACGTTGTGGAAAGAGCTGTCGTCTGAGATGGCTGAACTATCTCAGGCCTCATATTAAGCTTGG

AGGTTTCACTGAAGAGGAAGACAAAATTATCTGCAACCTTTATGGCACCATTGGAAGCAGGTGGTCCCTC

ATAGCAGCTCAACTTCCCGGGAGAACAGACAATGATGTCAAAAATCACTGGAACACCAAGCTAAAGAAAA

AGTTTCTAGCAGGAAACACTGGTAGTAGTACTGTTGCCACAAGCTACAACAATGGCACTGTCAGCAGTAC

TAGTTCTTCTGACCAATTCTCATCTTCTACTATTCAACCTCACGTTGAGGCCTTTGTGTTGAATCAGAAA

CTGATGAACTCAGCTTGGTTTGACTCCTACAATATTTTGGATCTGGAAAAAACATCAATTTCAGTTCCTC

TTCCAATGCCTCAGGAATTAGTATCATTTAATTATAGTGCACCACCCTCTAATGAAATTTCATTGCCAAC

TTTCAATGCAACTTCATTGGCACAGCAGAATGAAGATACACAATGGTTTGGCTATGACTATGCAGAGGAA

GATGATCCAAATTTACTTGCATTTGTGCTTGACGATCTGCTGATGAATGATGGGTCAGCATCTTCTGATC

ACATTGCTTCGTCATGTGTGGCTAACCAAGACTCGTTCTACTCATCAAAAACTTGAAAGAAAGAAAAAGG

GAAATTGTGGAATGGAATGACAGATTAAATAGCTCAAATCTGGGTAAAGTAGTTGTTGTATGTGGAATTG

TGTGAGCTTCTGAATTGTTTTGAATTGTGGTAGTATTATTAGAAGT

>XM_007135404.1 (PHAVU_010G131900g) RLM1A-like

ATGTGTTTCCGAGTTTCTCATAGAATCAAATATGTTGTATTCATTTGTCTGGTGTTTGTAACATCTTCTA

CTTGTTCCAATCACACTACTCAAATCAAGTACGATGTATTTGTTAGCTTCAGAGGTGTAGATGTCCGTCG

AGGTTTCCTTAGCCATTTGATTGAGGCATTTTCTCAGAAGCAAATTGCTTTCTTCGTAGATGATAACATT

CAGAAAGGGGAAGAACTATCTGAAGCACTATTAGGAGCCATTGAAGAATCATTAATTTCTTTGGTTATAT

TTTCAGAAAATTATGCTTCTTCAAGATGGTGTTTGTTAGAACTTGAGAAAATTTTGGAGTGCAGGAGAAA

AAATGGACAAATTGTAATGCCTATTTTCTACGAAGTAGATCCTTCAGAGGTACGGCACCAAAGGAGGACT

TATGGAGATGCCTTTGTTAAACATGAAAGAAACTATTCCTTCACTACTGTGCAAAGCTGGCGATCTGCTT

TGATTGAATCTGCTAATCTATCAGGATTTCATTCACCAATTTTTCAAGATGATGCTGAACTTGTTAAAAA

GACAGTTAAATTTGTGTGGAGGACGTTGAATCATGTTCACCAAGTTAACTCCAAAGGGCTTGTTGGAATT

GGTAAACGGATTGCTCATGTTGAATCATTGCTGCAATTAGAAGCAACACATGTTCGCATGATTGGAATTT

GGGGCATGGGTGGTATTGGTAAGACAACTATTGCACAAGAAGTATATAATAAACTTTGTTTTGAATATGA

TAGTTGTTGTTTTCTGGCTAACATAAGGGAAGATTCAGGAAGACATGGAATATTCTCATTGAAGAAAAAG

CTTTTTTCAACATTATTAGGAGAAGAACATTTAAAAGTTGACATATCTAATGGGTGGCAGAAGTTAGTTG

ACAGAAGACTGCATCGCTTGAAGGTTCTTATTATTCTAGATGATGTCGATGACTCAGAGCAACTAGAAAT

CTTAGCTAGAACAACTTGGTTTGGATCTGGTAGTAGAATCATTGTGACAACTAGAGATAAACAGGTTCTT

GCTAAAGAGTTTGCTAATGTATACCAAGTTGAGGCCTTAAACTTTGATGAATCTTTGCGACTTTTCAATT

TAAATGCCTTTAAGCGAAAACATCTGCAAGCAGAGTACCAGGAACTGTCAGAGAAAGTGGTTGATTATGC

CAAAGGCATTCCGTTTGTTCTTAAACTTTTGGGTCACCGTCTTCATGGGAAAGATAAAGAGATATGGGAA

AGTCAATTAGAAGGACAAATGCACAACAAAAAGGTTTATGATATTATTAAATTGAGTTACAATGATCTTG

AGGAGGATGAAAAGAGGATTTTTTTGGATATTGCATGTTTTTTTTATGGGCTGCAACTGGAGGTAAAGTA

CATAGATTTTTTATTGAAAGATCGTGATTATTCAGTGGTTGCGGGATTGGAAAGGTTGAAGGATAAAGCT

CTTATCAGCATTTCTCAAGAAAATACTGGTAACGAGGCTATCAGAAGCATAGTCATCAACTTGCTGAGAA

TGAAGCAACTACATCTAAAGCCACAAGTATTTACTAAGATGAGGAAGCTCCACTTTCTAAATTTGTACAC

TGCAGGAACTCGTGATATTCTCTTCTGTGAACCGTGGGGCTTGTATCTTCCTCAAGGGCTTGAATCTCTG

CCAAATGAGCTAAGATATCTTGGTTGGACGAATTATCCTTTGGAATATTTGCCTTCAAAGTTTTCTGCAG

AAAATCTAGTAGAGTTGCATTTGCCATATAGCCGGGTGAAAAAACTATGGCAAGAAGTGCCGGATCTTGT

GAATATAAAGGTCCTTATCCTGCATTCATCCTCAAACATAAAGGAGCTGCTAGACTTTTCAAAGGCCCCA

AACCTTGAAGTAATAGATCTTCGATTCTGTGTAGGGTTGACCAGTGTTCATCCATCAATTTTCTCTCTCA

AAAATCTTGAGAAGTTAGATTTGGATGGATGCACGTCCCTCACAAGCCTTCGAAGCAACATTCACTTGGA

CTCTCTTCGTTATCTCTCCCTCTACGGCTGCATGGAACTAAAGAATTTCTCAGTGACCTCAGAGAATATG

ATAAGGTTGAATTTAGAACGCACCAGTATCAAGCAACTCCCTTCATCTATTGGATCTCAGAGCAAGCTTG

TAAAGTTAAATCTTGCATTCACTTACGTTGAGAGCTTGCCTGAAAGCATAAAAAATCTTGAAGGGTTGCG

ACATCTAGATCTACGATACTGTGAGAAGCTTAGGTCCCTACCAAAGCTTCCTCCATCAGTTGAAACACTA

GATGTCCGTGAATGCATATCACTGGAGAGTATAACGTTCCCTCCTATTGCTGAACAATGGAAGGAAAATA

AGAAAAAGGTTGTCTTTTGGAACTGCTTTAAATTGGATGAGCATTCTCTCACGGCTATTGAGAGGAATGC

TGAAATCAACATGGTGAAATTTGCACACCAGCATTTGTCTACATCTGGTGATGCTCAAGGTATTTATGTG

TACCCAGGAAGCCAAGTTCCAGAATGGTTGATGCATAAGGCCACACATGATGATTACATAACTATTTCTC

CACATTCTTCTCACTTGGGCTACATCTTTTGCTTCATTTTACCGGAAGTGCCGAATGGGGAAAGGGTCTT

GAAATTGAAGATTAGTACTGAAGGTGAAGATGAAGGTGAAGATGACAGTATGATTGTGTACTTGGATAGA

CCACATCATGGGATTAAGTCGGATCACGTGTATCTGATGTACAACCAAGCATGTTCTCGCTTTCTACAAA

GTCGTGCCAAACATCAACGGAGGTTGAAAATTAAGTTTACAGTAACATCACTAGCACTCTCATCCGAATA

CATAAAGGTGCAGTTAAGAGGGTTTGGGGTGAGCACAACCAGCAATTTTCTTCAGAAACAAGAATTGGGT

GATGCTGGAATTCCAAAGGAACATTAA

>XM_007135465.1 (PHAVU_010G136800g) At4g11170

GAAAGTAATTAGCAGAAGTCAAAAGCGATCAAAATGAGAGAACCCCAAGAACATAATCCTTGAAAAGCTT

CATGGTCCATTTGTTTCATACGATGTTGTGATGTTGGTTGGTTCCTTCCTTTCCTCGAAAGGACAGTGGA

GAATATGCAAAGTTCGGAGATGTGTTCCCCCTTTGGCGAAAAGTACGATGTTTTTGTGAGTTTCAGAGGA

GCAGACACACGAACAAACTTCACAAACCATCTTCTGAATGCTCTGACTCAGAAATCAATCAGCGTATTCA

TAGATTACGAACTCACCAAAGGAGACTACATTTGGCCAGTCCTTGCCAGAGCAATCGAGGGGTCTCGTGT

GTCAATTGTTGTTATATCCGAAAACTATGCCTCCTCCAGCTGGTGCTTGAAAGAGCTGGAACACATACTC

CAATTCAGAAGAACTCGGGGTATGGTTGTTATACCCGTCTTCTATGAAGTAGATCCCTCACACGTAAGGA

AGCTGAGTGGGAGTTTTGATAAGTCTTTCGCAAAGCATGAGCTAGACACCGGATCATGTTCATGGGACCC

AGAAGCCCACAGGAAGGATGTTTCTAGGTGGAAAGCTGCTCTCAAACAAGCAGCCAATATTTCTGGATGG

GACTCCAGATCATACAGAAATGAAGCAACGTTCATCCAGAATCTTGTCAATGATGTATTGCAAAAGCTAC

AGCAGAGATATCCTACAGATCTAAAAGGCTTAGTCAGCATGAAAAATACTTGTGGAAAGGTGGACTTGTT

AGTGCAAAAATATCGAGTCATTGGAATTTGGGGTATGGGGGGAATTGGTAAGTCAACCATTGCTAAAGCC

TTGTTTGCCAAATACTTTCCCTCTTTTGATCATGTCTGCTTCGTGGCCAATGCAAAAGAATTTTCGCTCG

ATAAGCTTTTCTCTAACCTATTCAGAGAGGAAGTTTCCGCATCAAATGTGGTAGGATCAACATTTGATAT

GAGGAGACTCAGAAGGAAAAAGATTTTGATTGTACTTGATGATATGGATCGCTTAGACGTATTGGAATAT

TTATGTAGAGAGTATGAGGACCTAGATCCCGCTAGTAAACTCATCATAACAACACGAGATAAGCAATTGC

TCGAGGGAAGAGTTGATCAGATCTATGAGGTCAAGAAATGGAAAACCAGAGCATCTTTAAAGCTTTTTTG

CTTTGAAGCCTTCAAGCAAAAACATCCAAAAAAGGGTTATGAGATTCTCTCAGAAAGTGCAGTTGAATAT

GCCGGTGGTGTTCCACTAGCTCTTAAAGTTTTGGGTTCATATCTTCGTTCAAAAGGTATCAATTTTTGGG

AAAGTACTATAAGAAAACTCAGCTTGTATCCTAACGAGAGAATTCAGAAAGTGTTAGAAGTGAGCTACAC

TGGATTACATGATCTAGAAAAGAATATATTTCTAGACATTGTATTCTTTTTCAAAGAAAAACAGAAAGAT

CATGTCATTAGGATACTAGATGCCTGTGGTTTTGAAGCAACTAGTGGGATAGAAGTTCTCGCAGATAAAG

CTTTGTTAACAATTTCAAATAGAAAGATAATACGTATGCATGACTTGCTGCAACAAATGGGTTTGGAGAT

TGTACGTCAAGAGAGTAGTGGGGATCCTGGTAGCCGTAGTCGATTGAAGGATAATGAAGCTAGGGAAGTG

ATTGAAGAAAACAAGGGAACTGATGCTATTCAGGGCATAGCATTAGATTTGTCTCAAATTAAAGGTTTAA

TTTTGCATGCTGACACATTCACAAAGATGAAAACCTTGAGATTTCTGAAATTCTACAACACATTGGGTCA

GAGTGCTAGAGATACATACCTTGACCTTCCTGGAACTATTGAGCCATTTTCTGATAAACTGAGGTACATT

GAGTGGATTGGTTACCCGTTTGAGTCTCTTCCATCACCTTTTTGCGCTAAGGTTCTTGTTGAGATTCACA

TGCCACACAGCAAAGTAAAACAGCTTTGGCAGGGGATCCAGGAGCTCAATTATTTAGAAGGAATTGACCT

AAGACAATGCAAGCAGTTTGAGGAGCTTCCAGATTTGTCTAAGGCACCAAGACTTAAATGGGTGAATCTC

TCCTATTGTGAAAGTTTGCAGCATCTTCATCCATCTGTTTTATCCTCAGGCACACTTGTTACTTTGATAC

TGGATAAGTGTACAAACCTCAAGAGTGTTAAAAGTGAGAAACATTTAAAATCTCTAGAGAAGATCAGTGT

TAATGGCTGCTTAAATCTGGTGGAATTTGCAGTTTCATCAGATTTAATTGAAAATTTGGATTTAAGCTAT

ACAGGAATCCAAACATTGGACACATCAGGGAGTATGCATAAACTTAAGTGGCTCAATCTGGAGGGCTTAA

ACCTCAAGCATATTTTAGGAGAGTTATCTTGTTTGACATCTCTCAAGGTGCTGAAGATTTCGGGTAATGA

ACTGGTAATAGACAAACAGCAAATACATGTCCTGTTTAATGGATTAAGGTACCTGCAAATACTATATTTG

AAGGATTGTATTAAGTTGTTTGAACTCCCTGACAACATCAGTCTCTTGACACAATTACAGGAATTAAGGT

TAGACAGAAGCGATTTGAAAAGGTTGCCTGAAAACATTAAGAATCTTAAAATGTTGGAAATTCTGTCCCT

CGAGGATTGCAAGGAGCTTCTTTGCCTACCAAAGTTTCCATCCCTCATCAAACACTTACGTGCCATTAAC

TGTACATCATTGGTGTCAGTGACAAACTTGAAGACATTGGCAACAGAGATGTTGGGAATGACTAAACGCA

TTACATTCAAGAATAGTGGGAAATTGGATGGACATTCACTCAGAATTATTATGGACAGCCTCCATTTAAC

AATGATGAGTGCTGCATATCACAATGTGTTAGTAAGATCATATGGGACATACAAAATTTGCAACTATACG

AGTGTTGAGCTCTGTTTACCCGGAGGGACTGTCCCTGAGCATATCCATTATCGATCCACTAAGTCCTCCA

TTACCATTGCACTTCCTTCGCGTTCTGAATTACTGGGCTTCATTTACTCAGTGGTTCTTTCTCCAATTGG

TGGAATGAAGACTGACGGTGCTAAGATATTTTGTAAATGCCACTTGCCAGAAGAAGGTATAAAGGCAACA

TGGCTATATGGTGATATCAGGGGATTGAAGTCGGATCATGTTTATGTATGGTATGATCCATTACATTGTG

ACAACATTCTCAGATATTATGAACTATCAAAGGTTTGTTTTGAGTTCTGTGTTGCAAATGATAAGGGGGA

AGTTGACGGTTCCATCTGTATTAAAGAGTGTGGGGTAGGCATTATAAATGTTTTAGAAGTGCATAGTGTT

TTACAAGAGTTGGACTTTGATTCGGATAAGAAAAAGAAGTTGGTGGAGGGAGTGGAGTTGGAATCAAAGC

TTGGGAATCAACAACGGGTTTGGAGTGAATCAAGCTCTTCTGATACCTCTGAGTCAGACTCAGAGATTAT

TGTTAAACCAGATTCAGCTGAAAAGGGTAAATCTCCTCACAAGGAAATCAAAACTGATTCCAGAACAAAT

GCACTCAAATACGGAAGTAATAAAGAAGCACCAGTTAAATCAGATGCAACATTGCACGAGACCGTGGGGT

CACATTTAGACAATGAAAATGAGTTTAGAGAAAAGTCAATCACACAATCAAAGGTAGTAGTTATGGTTGA

GCTAGAGAACACTGAATCGACTTGCAAGGAAACCCTGAGCACTGCTAAAAGAAGTCCAAAAGAAAATTCT

AGAGAGTCTACAGAAATTGTGGCCAATGAACATTTCACACCACTTCAAGCCTCTTCCCAAGGGAGCTCTG

AGCACTTGCATGATAGGTTAGACAAATCGAATAAGCAAGTTGTAGAAACTCATGATACAGATAAATTTGT

TACTGAATACTCATCCTTTGACCTTGAGAATTGCCTCCAACAGTTGGATGAAAATCCATTTGCAATCCTT

GACTTACTCTCTAATGAGCCATCTCCTTCACTGAAACAATCAGAAACTTGTGTTCAAAAGGTAGCTCAAG

CAAATGATGCTACAACCATCCTCAATGAGTTCCGAACCTTAGTGTTCTCAACTTCGTTGCTAAAGAAACT

TCCAGACCAGTCCTATCGGCAACAAATTGAAGAATCACTACACAAGCTTAACACTTATCGTAGAGAAATT

ACAGAAGAGCAAAATGAGGGTTTAGAAAAATTTATAGAGCTATACAATAAAGCTTCCAAAATTTCTCAAG

ATAAGATGTTAACCGAGGATGAACAAGCAAAACTGGCATCTGAGAAGAGGGAGTTGTATAACAAACTTCA

AGATTCTAAACTGAAAGTTCAACAGTTTGACACCACCATCTCAACTAGTAAGTCTGAGATTGAAAACCTC

CAAAAAAGGCAAAGGGAAATCCAGGAAGCTATAAAAAAGCTTCAACAAGAAAAGGAAGCCTTGGAAAAGG

AGAGGTCAACACTAGAGTTTTTATGCTCAGAAAAACAAACAAAGAAGAACGAAACACTTGAATCAGTGAA

GCATATCTCAACTTCTGTTGTTTACACAACCAAACAATTAAAAGAACTTGTAGAGAAAAGATTGAGTTTG

GTTTCAGCTTACGAAGACCTCAAAAAGCCATATCAAAGAATGAAGACAAAGCCCCCATTTTAGTCTTCTT

TCCTGAGGTTTTTTCTTTTGATGTAATTTTTGTTTGTATCATTCCATACATAAGTTTCTGGCTGTAAATA

AGTGTAGTATGTTCAAGATGATAGCTATTTGTGTAAAGTGAAATTTGTATGAATGATGTACGAGGTTTCA

ATTAGTATTTGACCTCAAGTTTTAATGTATTTTGTAGGGATTGATTCAAAATACTATGGTTGTTGATAAG

TCACAATGTTTAACTTACAAGTGCTATCACTCTTTTACACTATCCATACTAGCTAATGTTGAAACTTCTT

TTATGCATTTTTTCATGGGTTTTTCTTTAATAATTCGTGTTCACCATTGTTATCAAACCAAACAAATTTT

GAGTTCTAAACTCCATTACATTTAAAAAAAGCATTAGCATGTAGAATG

>XM_007151169.1 (PHAVU_004G028900g) TMV-N like

ATGGCTGCAATGTCATGTTCCATTGCATTCTCCTATGATGTGTTCCTCAGCTTTAGAGGCTCAGACACAC

GCCATGGTTTTGTTGGCAACCTTTACAAAGCTCTTCAGGACAAGGGAATCCACACTTTCATTGATGATGA

GAAGCTTGAGGGAGGAGAGGAAATAACACCTACCCTTATGAAGGCAATTGAAGAGTCTAGAATTGCGATC

ACTGTGCTCTCTCACAACTATGCTTCTTCATCATTTTGTTTAGATGAACTTGTTAATATCATTGGTTGTG

CTGAGAAGAAAGGCTTGTTGGTTTTGCCTGTTTTTTACAACTTAGATCCTTCTGATGTGAGACACCAGAA

AGGTAGTTATGCAGAAGCATTGACAAAGCATGAGGAAAGGTTCAAAGTTAAGAAGGAAAGTTTCAACCAT

AACAAGGAGAGGTTGGAGAAATGGAAGATGGCTCTGCATCATGTGGCTAACATCTCTGGCTATCATTTCA

AACCAGGACATGAATATGAATATGAGTTTATTGGGAGGATTGTTGAGTTGGTCTCTAGCAAGATTAATCG

AAGTCCTTTACATGTTGCGGATTACCCAATTGGACTAGAGTCACAAATGTTAGAAGTAATGAAACTTTTG

GATGTTGGAAGTGATGATAAGGGTGTTCACATGATTGGGATTCATGGAATTGGTGGGATAGGAAAAACAA

CACTTGCTCTAGCCATTTATAATTTGGTAGCTCACCATTTTGATGGTTTGTGTTTTCTTGAAAACGTGAG

AGAAAACTCAGACAAACATGGGTTACAACATCTCCAAAGCATCCTTCTTGCTGAGTTGGTTAAAGAAAAG

AGAATGAACATAACAAGTGTGCAACAAGGAATTTCAATGTTACAACATAGGCTCCAACAGAAGAAGGTTC

TCTTGGTAGTAGATGATGCTGACAAGCATGAGCAATTGCAGGCTATTGTTGGCAGATCTGACTGGTTTGG

TTCCGGGAGTAGAATCATCATAACAACTCGAGATGAGCAGCTGCTAGCATCTCATAAGGTTAAAAGAACA

TATGTGGTGAAGGAACTGAACAAGAATGAGGCTCTTCAATTGCTTACATGGAAAGCTTTTAGAACTGATG

AAGTTGATCCAAGTTATGAGGAGGTCTTGAATAGTGTGGTAGCTTATGCTTGTGGCCTTCCATTGGCTTT

GGAAGTAATAGGCTCCAACTTGTTTGGAAAAAGTATAGAAGAATGGAAATCTGCTATCAAACAATATAAA

AGAATTCCTAATAATCAAATTCTAAAAGTACTTAAAGTAAGCTTTGATGCTTTGGAGGAAGAAGAGAAAA

GTGTTTTTCTTGACATTGCTTGTTGCTTCAAAGGATATGAATTGGAAGAGGTTCAAGATACACTTCATGG

TCATTACGGTGATTGCATGAAGTATCATATTGGGGTGTTGGTTGATAAATCTCTCTTAAAGCTTAGTCTG

AATGGTATGGAGGTGACAATGCATGACTTGGTAGAGGACATGGGTAAAGAAATTGTAAGAAAGGAGTCAC

CAAAAGATCCAGGGAAACGTAGCAGATTATGGTTGCATGAGGATATAATTCAAGTTTTAGAAGACAACAC

TGGAACTAGAGAAATTGAAATCATGCGTCTGGATTTTCCCTTACTTGACAAAGAAGGAATGATAGAATGG

AACAGAAAGGCCTTCAAGAAGATGAGAAACCTCAAAACACTAATTATTAAAAGTGGTAATTTTTCCAAAG

GTCCCAAATATCTTCCAAATAGTCTAAGAGTATTAGAATGGTGGAGGTATCCTTCACATGGTTTACCATC

CGATTTTCTTTCAAAAAAACTTGTCATGTGCAAGTTGCCTGAGAGTTGCTTTACATCACTTGAGTTGGTT

GACTTATTGAAGAAGTTCATGAGTATGAGATTTTTAAATCTGGACAAAAACAAATATTTAAAACAGATAC

CTGATGTATCTGGTCTCCCAAATTTAGAGAAACTCTCATTTCAACATTGTCAAAATTTAACTACAATTCA

CAATTCCATTGGATTTTTGCATAAATTGGAAATTTTGAATGCTTTTGGTTGCAGCAAACTTGTAAGTTTT

CCACCTATCAAGTTGTCTGCCCTTGAAAAGCTCAATCTCTCACGTTGTTATAGTCTTGAAAGTTTTCCAG

AAATATTAGGGAAGATGGAAAACATAAGGTCACTTCAGTTGGAGCACACTGCCATAAAAGAATTGCCATC

TTCTATTCAAAATCTTACACAACTTGAAGGGTTACAATTGTCTAACTGTGGAGTTGTTAAGTTACCAAGT

AGCATTGTTATGATGTCAAAATTGATTGATCTTATTGGTTGGAAATGGAAAGGGTGGCAATGGATAAAAC

AGGAAAAGGATGAAGAAAAAGAAGGATCATCTATAGTATCTTCAAATGTAGAATGCCTTTGGGTCTCAGA

ATGCAACCTGTGTGATGATTTCTTTTCAATTGGTTTCAAGCGATTTGCTCATGTGAAAGACTTAGACCTA

TCAAAGAATAATTTCACAATCCTACCTGAATGCATCAAAGAGTTTCAATTTTTAAGGAAGCTTAATGTGA

ATGATTGCAAGCTTCTTCGGGAAATTAGAGGGATTCCACCAAGCTTGAAACATTTCTTAGCAACAAATTG

TAAATCCTTGACTTCTTCAAGTATAAGCATGTTCCTAAATCAGGAACTACATGAGGCTGGAAAAACTCAA

TTTTATTTACCAGGAGAAAGGGTTCCGGAATGGTTTGATCACCAGTGCAATGGACCTTCAATTTCTTTTT

GGTTTCAAAATAGGTTCCCTAAGAAGGTTCTTTGTCTTATAATTGGACCTATTGCAGATGACTGTGGAAT

GTTTTTGCCTATGGTAATCATCAATGGCAACAAATGTTTTCGTGGTAGTGACCATTACATGATAGGAAGG

GATCATACATATATTATTGATATTCAAATGATAGGATTTGAAGAAAATTTATATGAAATTCCTTTTGAAA

ATGAATGGAACCATGCAGAAGTTAAATTTGTAGATTCGGAAGAGACAGCAATTCTTAAAGAAAGTGGAAT

TCGTATTTTCAAACAAGAAAGTAGCATGAAGGATATTTGGTTTTCTGATCCTTATGGCAAGAGAAAATTA

GAAGATAATCTCAATAGCTTGAAATCACAAAGCCAACAATTGCTAAAAAAGCATAGGTTTGTGGACATGG

AAGATTTGTAG

>XM_007151272.1 (PHAVU_004G037500g) RPK2-like

AGAAAATATTTTTTACATTAAAAAAAGACAAAAGGGAGAAGAAACGCGTTTCACAAAGAGAACAGTGCTG

AGTTGTGTCTTGTTCTTCCTTCTTCTCCATCGTTTCCATCATCGTCTTTCTCTCTTGTCATTACCCTTTC

CTCTTCTCTTCAACTTCACTCCAACACCACACTTCATTCTTCTTTAACTGTGAGATGTGTTCTGCTTCTT

CTGTTACTTCTTCTTCTTCTTCATCTTTTTCTTCATCTTCATCTTCTTCTTCTTCGTCTTCTTCTCCTTC

TTCTTCTCATTGCGGTTCAGTGATCAAAAGGAATACCCTCATGCAATTTCAATTCTTTGTCTTCGTGGTC

CTCCTCACGTTGCAAAACGACGCCGTTGCGATCGATTCGGACAAATCCGCGCTCCTCCGTATAAAGGCGT

CGTTTTCCGACCCCGCCGGCGTTCTCTCCACGTGGACCACCGCTGACAGTTCTGACTCCGGCCACTGCTA

CTGGTCCGGCGTCCTCTGCGACGCGAACTCCCGCGTCGTCGCCGTCAACGTCACCGGAAACGGCGGCAAC

CGAGCCTCGCACCCGTGCTCCGATTCCTCTAAATTCCCCCTCTACGGTTTCGGAATTCGGCGAACATGCA

AAGGGTTTTGTCTCTCCCCTTCAACGCGTTGGAGGGGGAAATTCCCGAAGCAATTTGGGGCATGGAAAAG

CTAGAGGTTCTCGATTTAGAAGGGAACTTGATAAGTGGCTATCTTCCCTTGAGAATTAATGGTTTGAGGA

AGTTGAGGGTTCTGAATCTTGGGTTTAATAGGATTATTGGGGAGGTGCCTAGTTCAATTGCGTCTCTTGA

GAGTTTGGAGGTTTTGAATTTGGCTGGTAATGAATTGAATGGTTCTGTGCCTGGTTTTGTTGGGAGGTTT

ACAGGGGTGTATCTTTCGTTTAATCAGTTCAGTGGGAATGTTCCGCCAGAGATTGGGGAGCATTGTTGGA

AGCTTGAGCATTTGGATTTGTCTGGGAATTCGTTGGTTCAAGGGATTCCGGTGAGTTTGGGGAATTGTGG

GAGGTTGAGGACGCTTTTGCTGTATTCTAATTTGTTGGAAGAAGGTATTCCTGGTGAGCTTGGGAAGCTT

AAGAGACTTGAGGTGTTGGATGTTTCCAGGAACACTCTCAGTGGCTCTGTGCCGAGGGCACTTGGGAATT

GCTCAGAGTTGTCGGTTCTTGTGCTGTCAAATCTCTTTGATGTGCGCGGGGATGCTGCCGGTGATTTTGG

GAAATTGGGTTCAGTGAATGATGAGGTGAATTATTTTGAAGGGTCAATTCCTGTGGAGGTTTTTTCGCTT

CCAAAGTTGAGGATACTGTGGGCTCCCATGGTGAATTTAGAAGGCAGTTTTAAGGGGAATTGGGGTGGTT

GTCAGAGCTTGGAGATGGTAAATTTGGCTCAGAATTTTTTCAATGGGGAATTTCCGAACCAGCTTGGTGT

CTGCAAGAGACTGCATTTTCTTGATTTAAGTGGAAACAATCTTACTGGGGTGCTTTCTGGAGAGCTTCAC

GTTCCCTGTATGAGCGTGTTTGATGTTAGTGGGAACATGTTATCTGGTTCAGTTCCTGATTTCTCCAATA

CCGATTGTCGCCCTGTTCCTTCCTCGAACGGAGACCTGTTTGAAGATGGGAATGTTTCCTCGCCATATGC

GTCGTTCTTTTTGTCAATGGTTCTTGAAAGATCTCTTTTTACATCAATGGGGGGAGTTGGTACTTCCGTT

GTTCACAACTTTGGGCAAAACAGCTTTACTGGCATTCAGTCGCTACCCATACCACATGACAGGCTGGGGA

AGAAGAACGGTTACACGTTTCTTGTTGGAGGAAATATTCTTACAGGATCATTTCCTACATATTTATTTGA

GAAATGTGATGGATTAGATGCATTACTTTTAAATGCCAGTTATAATAAGATAACTGGTCATATACCTTCC

AATATCAGTCGAATGTGCAGATCATTGAAATTTTTGGATGTGTCTGGAAATCAACTTGCAGGAACGATTC

CTGTTGATTTAGGGAATGTGGTCTCCCTTGTATCATTGAACCTCAGTAGGAATCAGTTGCAAGGTCAAAT

TCCCACCAGCCTTGGCCAGATGAAGAATCTAAAGTTTCTCTCTTTAGCTGGTAATAAGTTAAATAGCTCA

ATTCCTACCAGCCTGGGGCAGTTGTACTCTTTGGAAGTCTTTGACCTTTCTTCAAACTCTCTTACTGGTG

AGATTCCAAAGGCTATTGAGAACATGAGAAACCTGACTGATGTTTTGCTCAATAACAACAATCTTTCTGG

TCACATTCCTGATGGTTTGGCATATGTCACTACACTCTCAGCATTCAATGTGTCTTTCAACAACTTATCT

GGATATTTGCCTTCCAACAGTGGCTTGTTTAAATGCAGCAGTGCTGTTGGGAATCCGTACCTAAGTGCCT

GCCGCGGAGTCTCTCTGACTGTGCCATCAGGGAATCAGCTAGGGCCGATTGATAGCAACTCTTATAATAC

GGAAACAGAACAAGATACTGGCAAGAAGAGTGGGAGTGACTTCAGTTCTATTGAAATAGCATCTATAACT

TCTGCTTCAGCCATTGTTTCGGTACTTATAGCCCTGATTGTTCTATTCTTTTACACACGGAAGTGGAAGC

CAAGGTCCAGGGTTGTTGGCTCTACAAGAAAAGAAGTAACAGTGTTTACTGATATTGGGGTCCCATTGAC

GTTTGAAACTGTTGTCCAAGCCACAGGAAATTTCAATGCTGGCAACTGTATTGGGAGTGGAGGTTTTGGG

GCAACATACAAGGCAGAGATATCATCAGGAATCCTGGTGGCAGTCAAACGTCTAGCAGTTGGACGTTTCC

AAGGTGTTCAACAATTCCATGCCGAGATCAAGACCCTTGGGAGGCTTCATCATCCAAATCTTGTCACTCT

GATTGGTTATCATGCTTGTGAGACAGAGATGTTTCTCATATACAATTATTTGCCAGGTGGAAATCTCGAA

AAGTTTATCCACGAGAGGTCAACGAGGGCAGTAGACTGGAGAATTCTTCACAAGATTGCATTGGACATAG

CCCGTGCACTGGCCTATCTGCATGATCAGTGTGTTCCCCGTGTTCTTCACCGCGATGTCAAGCCCAGCAA

CATCTTGTTGGATGATGATTTCAATGCTTATCTATCGGATTTTGGATTGGCCAGACTTCTGGGAACTTCA

GAGACACATGCAACCACTGGTGTAGCAGGAACATTTGGGTATGTTGCTCCAGAATATGCAATGACTTGCC

GTGTTTCTGATAAGGCTGATGTGTATAGCTATGGTGTGGTGCTTCTGGAGTTGCTCTCAGACAAGAAGGC

ATTGGACCCTTCATTTTCTAATTTTGGAAATGGGTTCAACATAGTGGCATGGGCATGCATGCTACTGAAG

CAAGGAAGGGCAAATGAGTTTTTCACTGCCGGGTTATGGGAAGCAGGACCTGGAGATGATTTGGTAGAGG

TGCTTCACTTGGCAATTGTGTGTACTGTTGACTCTCTCTCTACCAGACCTACAATGAAACAAGTTGTCAG

AAGGCTTAAGCAACTTCAACCTCCGTCATGCTAGCCACTTCTGTGGCTTCTTCATCTTTAAACATTATTA

ATCTTAGAAACATTGTAATTTGTAATTTAGCATTTTTGTGGATTTAGGTTGAGTTCTCAATTTGTACTTA

TTCCCCCCCTCTGTACATTTTTATATAGTCGTGGCCCAATTTTGCATACTCTTTTCTTGTTGATTTGGTG

ATAGTTGGCTTGGTGTTGACTGTTGAATCTGCAGAAGAATGCAGATATTTGATTTTCC

>XM_007133236.1 (PHAVU_011G167800g) LRR receptor-like serine/threonine protein kinase At1g53430

CTCCGACAGACAAGCAGGAACAAAGCATCAAAATTTATGTCACAACCGAATGCGTGTGTTGACTGCGGAT

TAACTCATCTCACCAAATCGAAAACCATTATTGTTCCTTCTAACTTTTCTTTACCTTCTTATTTGTTCTC

CTTCAAGTGATATTGGTAAAAAATATCTCAATTTTCAAGGCCTCTCTCTTCAAACAAACAGATACAAACA

ACCACAAGACATACAAATCCTACTAACCACTCCTTTTGTTTTCCTAAAGTGTTATCATTACACAGTGGTC

GGTTCTGTCTCAAACCCAGCGAGTTTTCAAGCTTTGAGACAGCAACATGCGGTTAATCCATGTTCTTGTC

TTAGGGTTTGTGGCTCTTCATGTTTTTGAATCCAATGCTCAACTCATACCACAAGATGAAGTGAAAGTAC

TGCAAGCAATATCTGATAAACTAGAGAACTTGAATTGGACAGTTACCGAGCATTCCTGCGCAGAAGATGG

AGGGTTCGGTGACAGGATTAATATAAGGAATGACACCGTGAGGAATGTCTCATGCGATTGCAACTTCCAA

AATAACACAGTTTGCCATGTTGATAGCATCTTTCTGAAGGCTCAAAATATAGCTGGAGTTTTTCCCAGTG

AATTTGGAAATCTCACTCATCTGAAAGTGCTTGATCTAACACGCAACTATATCAACGGCTCGCTTCCAAA

AAGTTTTCCACCCAATGCTACACTCACCAATTTGTTACTTCTGGGAAACCGTCTAAGTGGTCCAATTCCC

TCAGAAATTGGTGATATTGCTAATTTGGAGGAACTGGTCTTAGATTGTAACCAACTTGAAGGACCACTTC

CTTCTAGTCTTGGAAATTTGAGCAACTTGAAGAGACTGCTTCTTTCTGCAAATAATTTTACAGGGACCAT

ACCAGAAACATTTGGAAAACTTGAAAATCTGACTGATTTTAGGATAGATGGAAGCAGTTTATCTGGACCA

ATACCCAGTTTCATTGGAAACTGGACCAAACTTAACAGACTGGATTTGCAGGGAACAAATATGGAAGGCC

CAATTCCTTCCACTATATCTCAGTTGAAACTTTTGACTAATTTGAGAATAACAGATTTGAAAGGATCAGC

AACTATGAATTTTCCTGATCTGGAGGAGTTGAAAAACATGGAGCGACTGGAATTAAGAAATTGCTTAATC

AAGGGTGACATTCCAGATTACATCGCTCAAATGTCATACTTAGACACTTTGGACCTAAGCTTCAACATGT

TAACTGGTCCAGTCCCAGATTTTATACCAAATTTAGGGAAGCTAGATTACTTGTTTCTGACAAATAATTC

GCTGAGCGGAGAAATTCAGGACTGGATACTGAGCTTTAAACATAACATAGATTTATCTTACAACAATTTT

ACTAAGTCTTCTGCATCTAGCTGCCAGCTCTCTGATGTGAACTTGGCTTCAAGTCATTCTTCTTCTTCAG

TGGCAACTACGGCTTCAACTTTTTGTTTGAAGAGGAACCTTCCTTGTGCAGGACAACCCCAGTATAAGTC

ATTGTTCATAAACTGTGGAGGAGGTGAGGGGGAGTTTGAAGGCAATAACTATGTAGGTGACCTCCATCTA

AATGGCATTTCAAACTTTGATCTTAGAAGTGAAGAACAATGGGCATATAGCAGCACAGGAGTATTTATGG

GAAATTATAGTGCAAATTATGTAGCAGAAAATGTATTTTCTTTGAACATTAGTGGTCCAGAATATTACCA

GAACGCTCGCCTTTCCCCTATGTCGCTTAACTACTATGGCCTTTGTATGCCAAACGGCAACTATAAAGTG

AAGCTCCATTTTGCCGAGATAATGTATTCTGATGATCAAACTTATAGAAGTCTAGGAAGGCGCATATTTG

ATGTTTCAGTTCAAGGTTTTAGATATCTGCAAGACTTTAACATTGTGGAAGAGGCTGGTGGAGTTGGAAA

GGGCATCACTAAGGAATTTGATGTTGATGTTCATGATGGTACCTTGGAAATCCACTTATACTGGGCAGGG

AAAGGAACTACTTCCATTCCTGATAGAGGTGTATATGGACCTCTTATATCTGCTATTGAGATGATACCAA

ACTTTGAGAATCCTTCAAAAGGGCTGTCTGCTGGAGTCATTGCTGGAATTGTTGCTGCATCATGTGGGTT

TGTCATATTGATACTGGTTGTCCTTTGGAAGATGGGTCTTCTTTGTAGGAAAGATACAACTGATAGAGAA

CTTCTAGATATGAAAACGGGCTATTACAGCTTAAGACAAATTAAAGGAGCTACTAATAACTTTGACCCTG

CAAATAAGATAGGTGAAGGAGGATTTGGGCCTGTATACAAGGGTGTGCTGTCAGATGGTGATGTGATTGC

AGTTAAGCAGCTCTCCTCCAAATCAAAGCAGGGGAACCGAGAATTTGTCAATGAAATTGGAATGATATCT

GCTTTGCAGCATCCAAATCTGGTGAAGCTATATGGTTGTTGCATTGAAGGAAACCAGTTGCTACTGATAT

ATGAATACATGGAGAACAACAGTCTTGCTCGTGCACTTTTTGGTGAAGAAGAGCAGAAGCTGCACTTAGA

CTGGCCTACAAGAATGAAGATCTGTGTGGGGATAGCAAGGGGACTGGCTTATCTTCACGAGGAATCAAGG

TTGAAAATAGTGCACAGGGACATTAAGGCAACCAATGTCTTACTTGACAGGGATCTGAATGCCAAGATCT

CTGACTTTGGTTTAGCTAAGCTTGATGAAGAAGAAAATACTCATATCAGTACACGTATAGCTGGAACAAT

TGGTTACATGGCCCCGGAATATGCTATGAGGGGTTACTTGACTGATAAAGCAGATGTATATAGCTTTGGA

GTTGTAGCTTTAGAGATTGTTAGTGGAAAAAGCAACACAAAATACAGGCCAAAGGAAGAGTTTGTATATC

TTCTAGATTGGGCTTATGTTCTCCAAGAGCAAGGAAACCTTCTGGAATTGGTGGATCCAAGTCTTGGTTC

AAAGTACTCTCAAGAGGAAGCCATGAGAATGCTGAGCTTGGCACTCTTGTGCACCAATCCATCTCCTTGA

CATTCTTAGGAATCATCTTGAGTTGGATATGAGCTAGACA

>XM_007133253.1 (PHAVU_011G169300g) LRR receptor-like serine/threonine protein kinase At1g56130

TTGGTGTGCCATGACAGAACCTGACGTAAGCTTTTGACATAGCTCAAGCTGAAAATTGAGGTCAGAGACG

CCATTTTTGTCTCTCTTTTTTCACAAAAGTCTTATAAATATAAAGTAGGGTTCCAATTTTTCAACCCAAC

ACTGCCGTTTCTCACCAAATACCATCGTTCTTATCATTCTGTGAGGTCGATTCCTCAAGTTTCAAACTAC

TACAAAACATTTTTTTAAAGATCGTTCTTCTGGGATAATCAGTTGTTCATATATCAGGATGTCACAAGGG

TTTGCTTTTGCACTTGTTGCTGTGTTCTTCTGTCACTTCAGCTGCTTTCTTAGCTCTGCACAAGCTCAAT

CTGCAAACGCCACCACTGACCCTTCTGAAGCAAGAACTATAAATTCAATCTTCAGCAAATGGGGTAAATC

CGCAGACACAAGTATATGGAATATAAGCGGTGAATTGTGCAGTGGAAGAGCCATTGATAGTACCTCCACC

CCTGAATCTTACAACCCATTTATCAGATGTGATTGTTCCTTTGACGACGGAACTACTTGCCGCATCACAG

CACTGAGGGTCTCTGCACTGAATGTGGTTGGTGAAATTCCAGAAGAGCTATGGACTCTCACCTATCTCAA

CAATTTAAATCTTGCGCAAAATTACTTGACGGGTTCTCTACCTGCAGCTATTGGAAATCTAACTCGTATG

CAATACTTGAGCTTTGGAATCAATAATTTATCAGGGGAGCTTCCAAAGGAATTGGGAAATCTTACAGAGT

TATTATCACTGAGTTTTTCGTCAAATAAGTTCTCAGGATCTCTCCCATCTGAACTTGGGAAACTCACAAA

GTTAACCCAGCTTTACATTGATAGTTCAGGAATTAGTGGTCCAATTCCGTCCTCATTTGCGGACCTAAGG

AGTTTGGAACGAGTATGGGCTTCAGACACGGAACTCAGGGGCAATATACCAGACTTCATAGGGAATTGGA

CTAGACTTCAAGTCTTGAGGTTTCAAGGCAATTCTTTTAATGGCTCAATACCCTCATCATTTTCCAACTT

GACTTCTTTAACAGAATTGAGAATAAGTGGTTTATCTGATGGGAACTCCTCACTGGAATTTGTACGGAAT

TTGAAATCTTTGAATATCTTAGAATTGAGGAATAACAATATTTCTGGTTCGATTCCCTCCTCCATTGGAG

AGTTGCACAATTTGACCCAGCTGGATTTAAGCTTCAATAACATCGAGGGACAAATTCCGGGATCAATTTT

CAATTTGAGTTCGCTCTCTACCTTGTTTCTTGGAAATAATAAGCTAAATGGCACCCTTCCAACGCAGAAA

AGTTCATCATTTCAATTTATAGACTTGTCATACAATGATCTATCAGGTAGCCTCCCCTCTTGGGTAAACG

ATGCAAATTTACAACTGAATTTAGTTGTTAACAACTTGACAATAGATGATGATTCAGATACCAGTGGCTT

GCCAAATGGGCTCAACTGTCTCCAGAAAAATTTTCCTTGCAATCAAGGTGTTGGAAGATATTCTGACTTT

GCAATCAAGTGTGGCGGTTCCCAAATTACGTCCACAGAAGGAATAGTGTATGAAACGGAGAATGAGACAC

TTGGTCCTGCTACATACTTTGTTACTGATACAAGTAGATGGGCTGCTAGTAATGTTGGATTATTTACGAA

CAATAATAATCCAAAATTCACAAAATCTGTGACTAATCAATTCACTAACACTATGAATTCGGAGCTCTTC

CAAACAGCACGACTCTCTCCTTCATCATTGAGATATTATGGCTTGGGGCTGGAAAATGGCTTTTACAACA

TCACCCTTCAATTTGCAGAAACAGCTATTGAGGATTCTACTACAAAATGGGAAAGTCTTGGGAGACGAGT

CTTTGATATATATATTCAGGGGAATCTTTTCTTGAAAGATTTCGACATACAAAAGGAAGCTGGGGGCATA

TCATTCAGAAGTGTCCAAAAGCAATTTAGGTTTGAAGTGTCTGAAAACTATCTTGACATCCATCTCTTTT

GGGCAGGAAAAGGGACTTGTTGCATACCAAATCAAGGTACTTTTGGGCCCTTGATTCAAGCCATCCATGC

TATCCCAGATTTTATACCTAGTGTCAGTAACGAACCTCCAAGCAATAAAACAAATAGAACTGGCCTAATT

GTTGGAATTGTTGTTGGAGTTGGAGTTGTATGCTTCCTATCAGTTTTTGTGACTTTTTGTATCATTCGGA

GAAGAAAACGTCAGCATGAGGATGAAGAGCTTTTAGGAATTGATACAAAGCCATACACTTTCAGTTATTC

TGAGTTGAAGAATGCTACTAATGACTTCAATATCGAAAATAAGCTTGGAGAGGGAGGTTTTGGACCTGTT

TATAAGGGGACACTTAATGATGGAAGGGTTATTGCTGTGAAACAACTGTCAGTAGCATCCCATCAAGGAA

AGAGCCAGTTCATAACTGAGATTGCTACTATATCAGCTGTGCAACATCGTAATCTGGTGAAACTATATGG

ATGTTGTATTGAGGGAAACAAAAAGCTTCTGGTGTACGAGTATCTGGAGAATAAGAGTCTTGATCAAGGA

TTATTCAGTAATTCTTTAACCCTCAATTGGTCCACACGCTATGATATCTGCTTGGGTGTTGCCAGAGGTT

TAGCTTATTTACATGAAGAGTCTCGTCTCCGTATTGTACACCGTGATGTGAAGGCTAGCAATATTTTGCT

TGACTATGAGCTTGTCCCCAAAATATCAGATTTTGGGTTGGCCAAATTGTACGATGATAAAAAGACCCAC

ATAAGCACTCGTGTGGCTGGAACAATTGGATATCTTGCACCGGAGTATGCCATGCGTGGACACCTTACAG

AGAAAGCAGATATATTTTCATTTGGTGTTGTTGCTCTAGAGTTAGTCAGTGGGAGACCAAATTCTGATTC

AAGCTTGGAAGGAGAGAAGGTGTATCTTCTGGAATGGGCTTGGCAGCTTTATGAAAACAACCTCTTAATG

GATCTGGTGGACCCTAGAATATCAGAATTCAATGAGGAAGAAGTAAAACGTGTTGTGGGAATAGCACTTC

TGTGCACTCAGACATCACCATCATTAAGACCATCAATGTCCCGTGTGGTGGGAATGCTTTCAGGAGATAT

TGAAGTGAACAGTGTCACTACAAAGCCTGGATACCTTACTGACTGGAAATTTGATGATGTGACCAGCTTC

ATGACTGAGAATGCAATTGAAGGATTGAATACAAGTCACCAGTATTCATCAGGAAGTACCAGCATTGTGG

GTGGTACAGACTTTACACCATTAAGTGTTTCAAAACTGAACCTTAATGATGGTCTTAGTGAGGGTAGGTA

AGAATTGGTGTACTCATTACTGTTCAGATCAAGTGTAGAACAATTATCATGTCACTGCTCCAAAACTTGT

TTTCTTTTTCTCTTGTTTTGCATTTTCTTTAGCCATTTGTTTTATTGTGTAGTTAATGTAAATAGCCCCC

TAAGTTTCAAGAGAATGTACAACAATGTGTCGCTAATAGGCCTCAACCAGGAAAGTTTATAGGGGATTTT

TTTGTATAAACACTATTTTCTTTGTTGTGATTTTTGTTTTGAACTACCCTTTTCTACAAATGAAGTCATG

GAATAAATTATTTGTTGTAATTGATAGCTGTTTGGAATTTAGAGTGTGAGATGTATTCAATCC
